# Isolated native pulmonary valve endocarditis on a bicuspid valve with large vegetation and septic pulmonary emboli: a multimodality imaging case report

**DOI:** 10.1093/ehjcr/ytag687

**Published:** 2026-09-21

**Authors:** Shabnam Boudagh, Marziyeh Najafi, Mohammad Reza Tolou Ostadan Yazd, Reza Khosravi

**Affiliations:** Rajaie Cardiovascular Medical & Research Center, Iran University of Medical Sciences, Valiasr Avenue, Niayesh intersection, Tehran 1995614331, Iran; Rajaie Cardiovascular Medical & Research Center, Iran University of Medical Sciences, Valiasr Avenue, Niayesh intersection, Tehran 1995614331, Iran; Department of Cardiology, Hajar Hospital, Shahrekord University of Medical Sciences, Parastar Avenue, Shahrekord 8816714811, Iran; Department of Pediatric Surgery, Children′s Medical Center, Tehran University of Medical Sciences, Gharib Street, Keshavrz Boulevard, Tehran 1419463151, Iran; Department of Cardiology, Hajar Hospital, Shahrekord University of Medical Sciences, Parastar Avenue, Shahrekord 8816714811, Iran

**Keywords:** Case report, Pulmonary valve endocarditis, Bicuspid pulmonary valve, Echocardiography, Computed tomography, Septic pulmonary emboli, Valve replacement

## Abstract

**Background:**

Isolated pulmonary valve endocarditis (PVE) is a rare form of infective endocarditis (IE), accounting for fewer than 2% of all cases. Its presentation often mimics primary respiratory disease, and visualization of the pulmonary valve may be challenging, leading to diagnostic delay. Multimodality imaging is therefore essential for early recognition and treatment planning.

**Case summary:**

We report a 59-year-old woman with a history of colorectal cancer treated with surgery and colostomy who presented with 1 month of fever and progressive dyspnoea. Transthoracic and transoesophageal echocardiography demonstrated a bicuspid pulmonary valve with two large, highly mobile vegetations (largest 3.0 × 0.9 cm), mild-to-moderate pulmonary regurgitation, and a small perimembranous ventricular septal defect with left-to-right shunt. Chest computed tomography (CT) showed multiple segmental and subsegmental filling defects compatible with septic pulmonary emboli and signs of right ventricular strain. Blood cultures grew group D viridans streptococci. Despite 6 weeks of targeted intravenous antibiotics, she developed right-sided heart failure with persistent large vegetations. She underwent pulmonary valve replacement with a 27-mm mechanical bileaflet prosthesis and concomitant repair of the ventricular septal defect and closure of the patent foramen ovale. Follow-up imaging confirmed a well-functioning prosthetic valve with improved haemodynamics.

**Discussion:**

This case highlights the diagnostic and therapeutic value of combining echocardiography and CT in native PVE. Multimodality imaging allowed timely identification of the underlying congenital substrate, precise characterization of vegetation burden, recognition of septic pulmonary emboli, and informed surgical decision-making in accordance with current guideline-based indications for right-sided IE surgery.

Learning points

- Isolated native pulmonary valve endocarditis is rare and may mimic primary respiratory disease, leading to delayed diagnosis.
- Multimodality imaging with transthoracic echocardiography, 3D transoesophageal echocardiography, and computed tomography pulmonary angiography is essential.

## Introduction

Infective endocarditis (IE) is a life-threatening infection of the endocardial surface of the heart that predominantly affects left-sided valves. Right-sided IE accounts for only 5%–10% of cases and most often involves the tricuspid valve in high-risk populations.^1,2^ Isolated pulmonary valve endocarditis (PVE) is the rarest manifestation, representing fewer than 2% of hospitalized IE cases.^1,3,4^

## Summary figure

**Figure:**
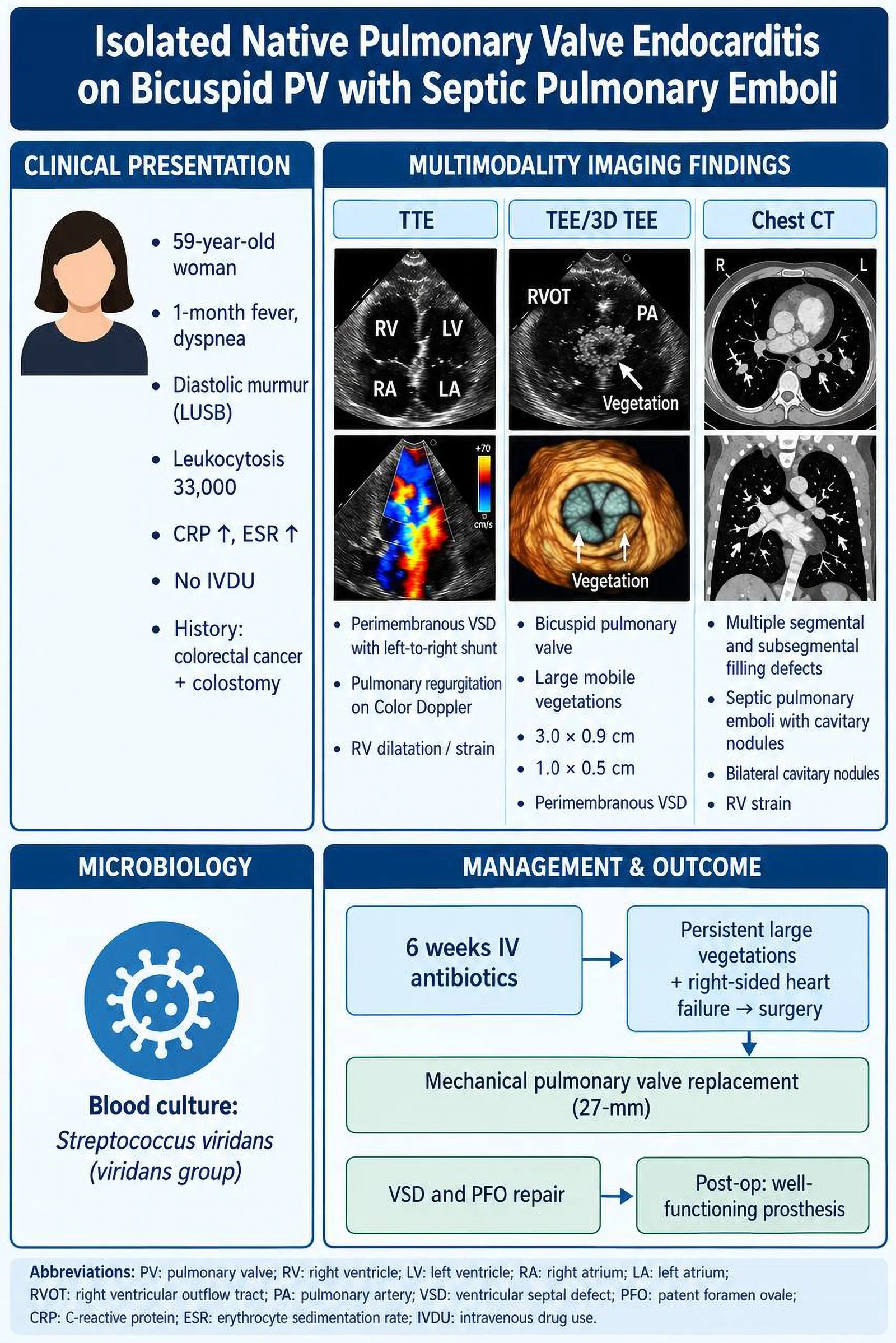


Several physiological characteristics of the pulmonary circulation, including lower pressures, lower oxygen content, and less turbulent flow, are thought to confer relative protection against infection of the pulmonic valve. When PVE does occur, it is often associated with congenital or acquired abnormalities of the right ventricular outflow tract, such as ventricular septal defect (VSD) or bicuspid pulmonary valve, or with systemic predisposing factors such as central venous catheters, chronic infection, or immunosuppression.^1–3^

The clinical presentation of PVE frequently mimics primary pulmonary disease, with fever, cough, dyspnoea, and radiographic infiltrates, which may delay diagnosis. Echocardiography remains the cornerstone of IE assessment, but visualization of the pulmonary valve can be suboptimal on transthoracic views.^1,5,6^ Integration of transoesophageal echocardiography and chest computed tomography (CT) can improve diagnostic accuracy and detect septic pulmonary emboli.^5,6^ We report a rare case of native PVE on a bicuspid pulmonary valve complicated by extensive septic pulmonary emboli, underscoring the role of multimodality imaging in guiding management.

## Case presentation

A 59-year-old woman with a history of colorectal cancer treated 3 years earlier by surgery and colostomy was admitted with 1 month of persistent fever, chills, and progressive dyspnoea. She denied intravenous drug use, recent invasive procedures, urinary or gastrointestinal symptoms, or local irritation around the colostomy.

On admission, she was febrile (39.5°C), mildly hypotensive (95/56 mmHg), and tachycardic (115 beats/min). Physical examination revealed a well-functioning colostomy without erythema or discharge, a diastolic murmur along the left upper sternal border, decreased breath sounds bilaterally more prominent on the right, and bilateral pitting oedema of the lower extremities. There were no peripheral stigmata of IE such as splinter haemorrhages, Osler nodes, or Janeway lesions.

Laboratory investigations showed leucocytosis (33.8 × 10^3^/μL, 90% neutrophils), microcytic anaemia (haemoglobin 7.8 g/dL), and markedly elevated inflammatory markers. Renal and liver function tests and the coagulation profile were within normal limits, while mild hyponatraemia, hypocalcaemia, and hypoalbuminaemia were present.

## Imaging, management, and follow-up

**Figure 1:**
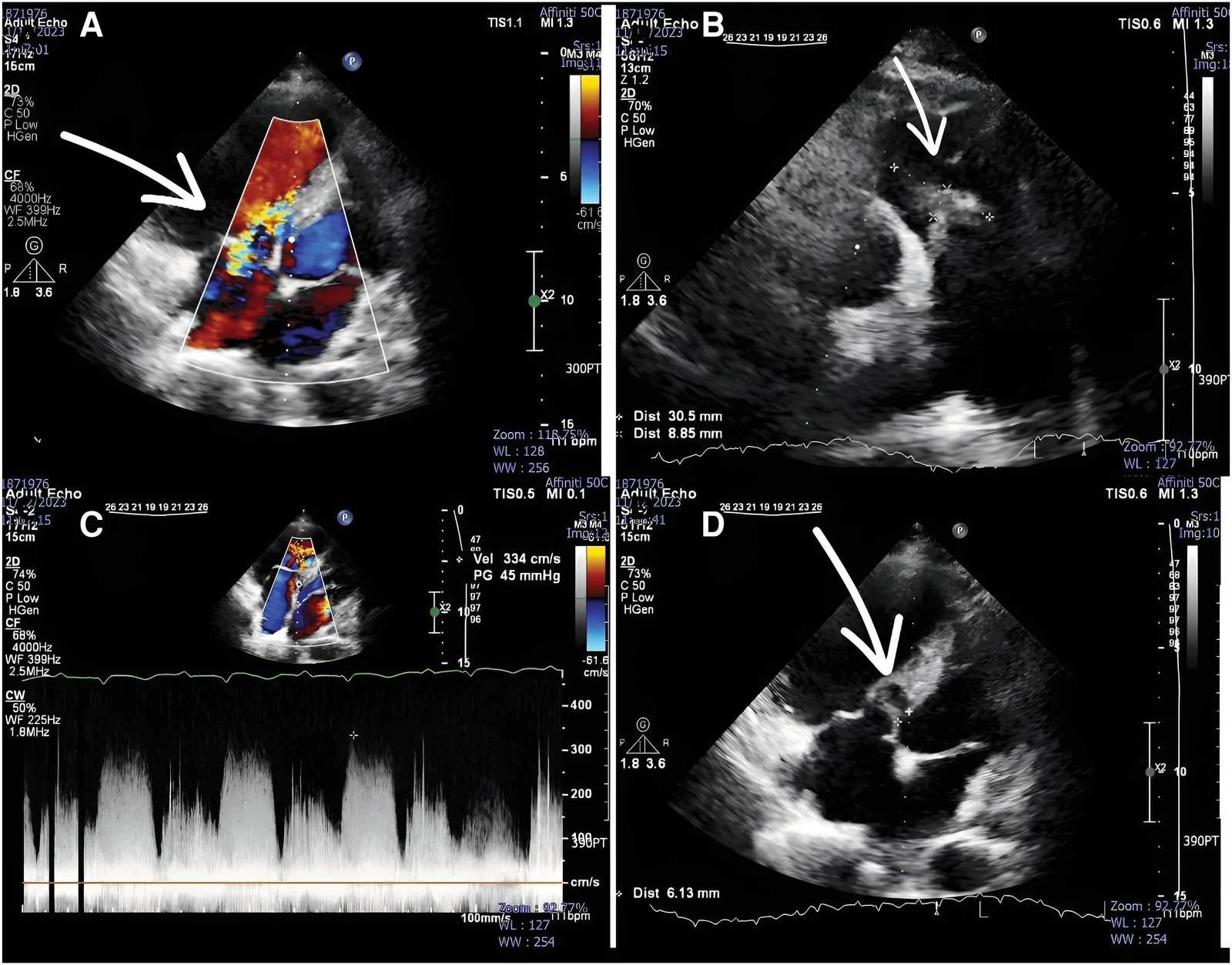
Transthoracic echocardiography (TTE) images demonstrating ventricular septal defect (VSD) and vegetations involving the pulmonary valve (PV). (*A*) Off-axis apical four-chamber view demonstrating a VSD with left-to-right shunt during systole. (*B*) Right ventricular outflow tract (RVOT) view showing the PV with multiple large, mobile vegetations attached to the valve leaflets. (*C*) Pulsed/colour Doppler interrogation of the VSD demonstrating bidirectional flow, with both systolic left-to-right shunt and diastolic flow across the defect. (*D*) Measurement of VSD size from the left ventricular (LV) side.

Transthoracic echocardiography demonstrated preserved left ventricular ejection fraction (50%–55%) with mild left ventricular hypertrophy. The pulmonary valve appeared thickened and myxomatous with mild pulmonary regurgitation. Two semi-mobile, shaggy, multilobulated echogenic masses were attached to the pulmonary valve leaflets, the largest measuring 3.0 × 0.9 cm (*Figure 1*). The main pulmonary artery and its branches were dilated. The tricuspid valve was thickened with at least moderate regurgitation, and a small perimembranous VSD (6 mm) with left-to-right shunt was present, partially sealed by the septal leaflet of the tricuspid valve. The inferior vena cava was plethoric, consistent with elevated right-sided filling pressures.

**Figure 2:**
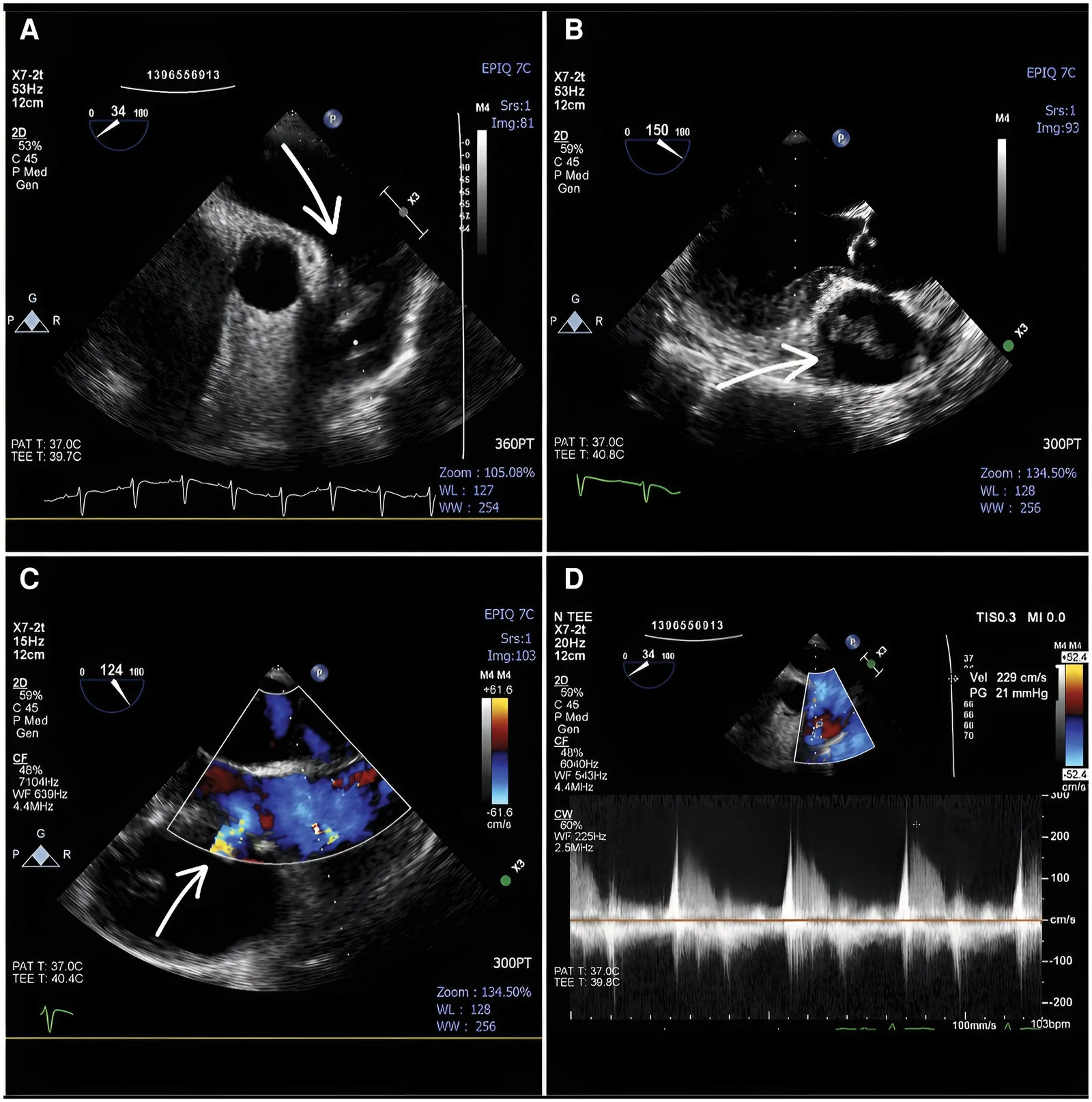
Transoesophageal echocardiography (TEE) demonstrating pulmonary valve involvement. (*A*) Mid-oesophageal TEE right ventricular outflow tract (RVOT) view showing a mobile echo-dense mass attached to the pulmonary valve, consistent with vegetation. (*B*) Additional TEE off-axis view confirming the presence and mobility of the pulmonary valve vegetation. (*C*) Ventricular septal defect (VSD) with left to right shunt. (*D*) Doppler study of moderate pulmonary insufficiency (PI).

**Figure 3:**
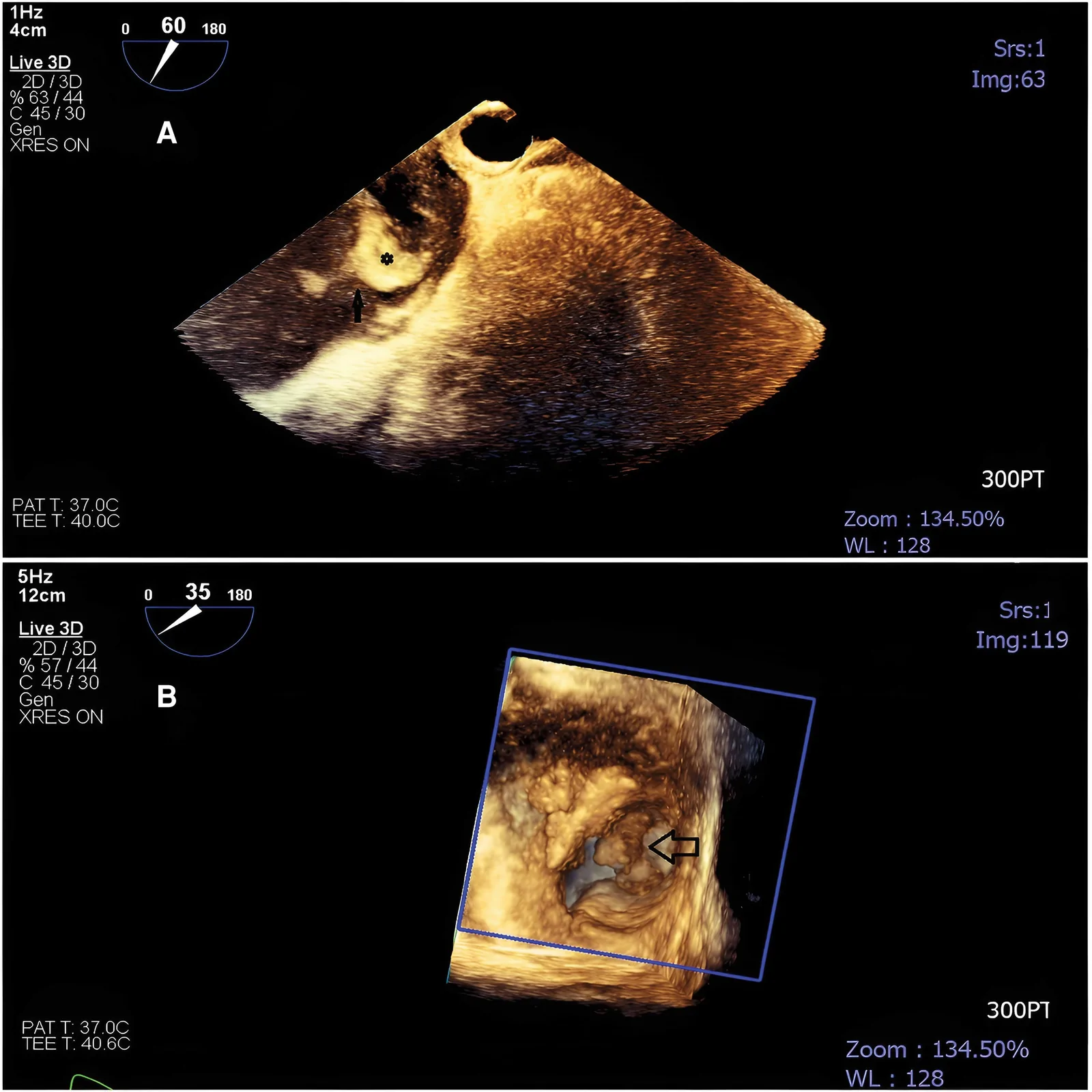
3D transoesophageal echocardiography (3D-TEE), (*A*) 60° en-face view and (*B*) 30° cropped view demonstrating a bicuspid pulmonary valve with multiple large, irregular, highly mobile vegetations (arrows) causing marked anatomical distortion.

Transoesophageal echocardiography was performed to better characterize the pulmonary valve and VSD in the setting of suspected IE (*Figure 2*). 3D imaging revealed a bicuspid pulmonary valve with two large, highly mobile vegetations attached to the cusps and associated mild-to-moderate pulmonary regurgitation, without vegetations on other valves or along the VSD margins (*Figure 3*). The severity of pulmonary regurgitation was assessed using an integrative echocardiographic approach in accordance with current guideline recommendations, including colour Doppler jet width and extent, continuous-wave Doppler signal density and deceleration, and supportive right ventricular size and haemodynamic findings.

Right ventricular systolic function was impaired on echocardiography, demonstrated by reduced tricuspid annular plane systolic excursion of 12 mm, tricuspid annular systolic velocity (S′) of 6 cm/s, and functional area change of 26%, consistent with moderate right ventricular systolic dysfunction.

Given the suspicion of right-sided IE with embolic complications, contrast-enhanced chest CT was obtained. CT demonstrated a peripheral filling defect was seen in the lobar branch of the right upper lobe, interlobar artery and also complete obstruction of subsegmental branches of posterobasal of right lower lobe, laterobasal, and posteromedial of left lower lobe (LLL) artery in favour of septic pulmonary embolism.

**Figure 4:**
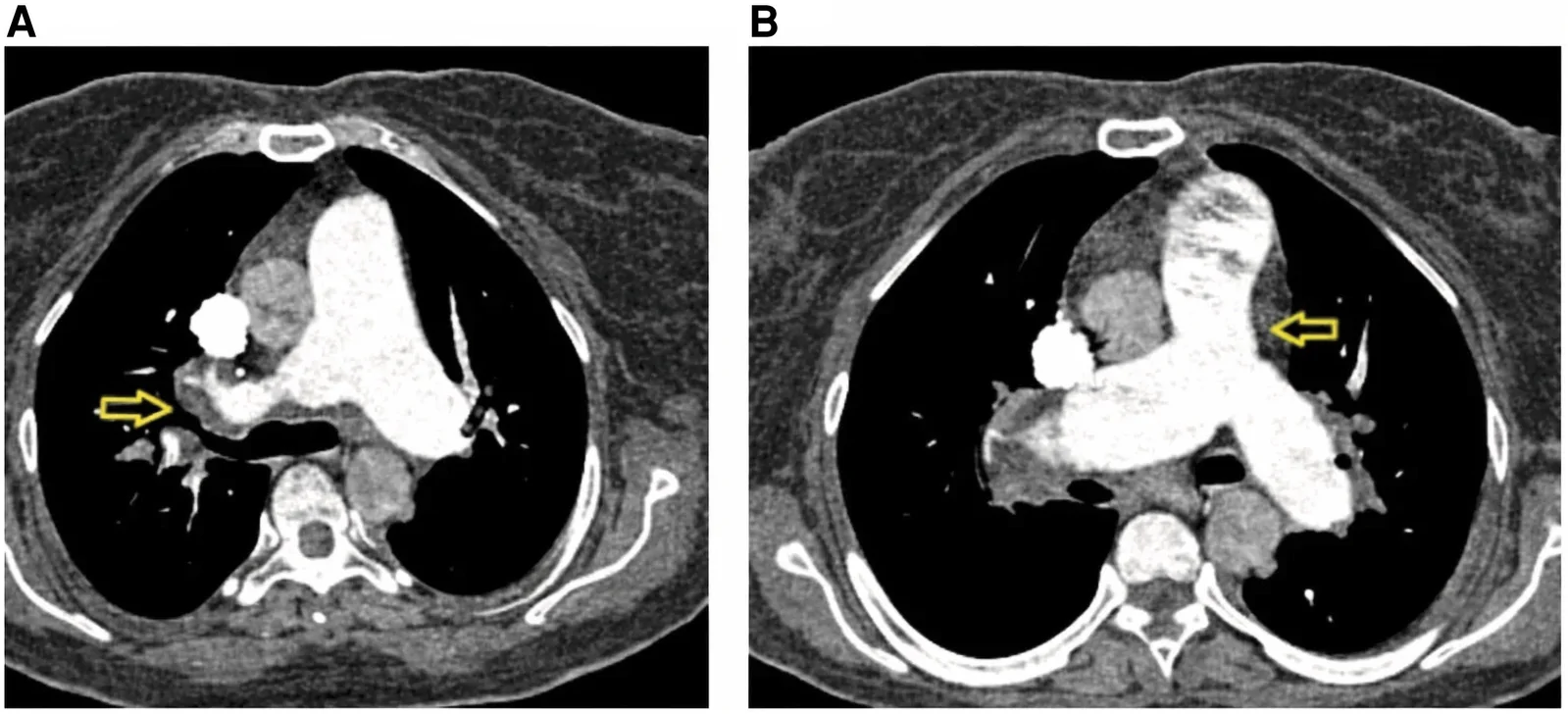
Contrast-enhanced computed tomography pulmonary angiography (CTPA) demonstrating pulmonary embolism. Intraluminal filling defects are visible within segmental branches of the pulmonary arteries (arrows). Panels (*A*) and (*B*) show embolic material in different pulmonary arterial branches, consistent with pulmonary embolism.

The main pulmonary artery, right pulmonary artery, and left pulmonary were enlarged in favour of chronic thromboembolic pulmonary hypertention (CTEPH). Right ventricular strain pattern is also evident. Pulmonary infarct in posterior segment of LLL was noted (*Figure 4*).

Abdominal and pelvic CT scans did not reveal any intra-abdominal source of infection or abscesses.

Empiric broad-spectrum intravenous antibiotics were started on admission and later tailored after blood cultures grew Gram-positive cocci identified as group D viridans streptococci. Targeted intravenous therapy was continued for 6 weeks. Despite appropriate antimicrobial treatment, the patient developed progressive signs of right-sided heart failure and repeat echocardiography showed persistent large vegetations on the pulmonary valve with ongoing pulmonary hypertension.

In view of the large (>20 mm) mobile vegetations, recurrent septic pulmonary emboli, and right-sided heart failure despite adequate antibiotic therapy, she was referred for surgery in line with current guideline-based indications for right-sided IE.^1,2^ She underwent pulmonary valve replacement with a 27-mm mechanical bileaflet prosthesis, repair of the perimembranous VSD and closure of the patent foramen ovale, and reconstruction of the pulmonary artery and right ventricular outflow tract. The postoperative course was uneventful, and she was discharged on long-term anticoagulation and antibiotic prophylaxis for future invasive procedures.

Follow-up transthoracic echocardiography demonstrated a well-functioning prosthetic pulmonary valve with a mean gradient of 8.7 mmHg and no paravalvular leak and also showed moderate tricuspid regurgitation. Right ventricular function remained moderately impaired but haemodynamics had improved, with a right ventricular systolic pressure of 42 mmHg. The VSD patch was intact without residual shunt, and no recurrent vegetations were seen.

## Discussion

Isolated native PVE is exceedingly rare and often under-recognized.^1,3,4^ Difficult visualization of the pulmonary valve and the predominance of respiratory symptoms contribute to diagnostic delay or misclassification as pulmonary infection or embolism.^1,5,6^ In our patient, the initial presentation with dyspnoea, fever, and bilateral cavitary pulmonary nodules could have been attributed solely to pneumonia or pulmonary embolism, highlighting the importance of maintaining a high index of suspicion for right-sided IE in such scenarios.

The underlying bicuspid pulmonary valve and perimembranous VSD in this case likely created abnormal flow patterns and endothelial disruption in the right ventricular outflow tract, predisposing to vegetation formation even in the absence of intravenous drug use or intracardiac devices.^3,4^ Structural abnormalities of semilunar valves arise from aberrant endocardial cushion development and are frequently associated with other congenital lesions. Recognition of these substrates is essential, as they can modify both risk and long-term management.

Multimodality imaging played a central role in establishing the diagnosis and defining the extent of disease. Echocardiography allowed identification of a bicuspid pulmonary valve, quantification of vegetation size and mobility, assessment of pulmonary regurgitation, and evaluation of right ventricular function.^1,5,6^ 3D transoesophageal imaging provided additional anatomic detail for surgical planning. Chest CT complemented echocardiography by demonstrating extensive septic pulmonary emboli and pulmonary infarction, thereby clarifying the mechanism of the patient’s respiratory compromise and right ventricular strain.^5,6^

The decision to proceed with surgery was based on several classical indications for right-sided IE: large vegetations, recurrent septic pulmonary emboli, and progressive right-sided heart failure despite appropriate antibiotic therapy.^1,2^ Current ESC and AHA guidelines support early surgery in such cases to prevent further embolization, control infection, and address haemodynamic deterioration.^1,2^ In our patient, valve replacement with simultaneous repair of associated congenital defects achieved effective source control and haemodynamic improvement. This case also illustrates the importance of a multidisciplinary heart team approach, involving cardiology, infectious diseases, radiology, and cardiothoracic surgery, to optimize the timing and type of intervention in rare presentations such as native PVE.

Histopathological examination of the excised pulmonary valve was not available, which represents a limitation of this report. However, the diagnosis was supported by concordant clinical, microbiological, and multimodality imaging findings, fulfilling guideline-based diagnostic criteria for IE. Although rare, the possibility of alternative or dual pathology cannot be completely excluded.

## Conclusion

Native PVE should be considered in patients presenting with unexplained fever, respiratory symptoms, septic pulmonary emboli, or right ventricular dysfunction, particularly when congenital abnormalities of the right ventricular outflow tract are present. Comprehensive multimodality imaging with transthoracic and transoesophageal echocardiography and chest CT is indispensable for early diagnosis, characterization of vegetation burden, detection of embolic complications, and surgical planning. Adherence to guideline-based indications for surgery and a multidisciplinary team approach are key to improving outcomes in this rare but serious condition.

## Lead author biography


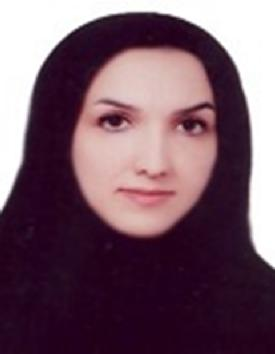


Dr Shabnam Boudagh is an assistant professor of cardiology at Shahid Rajaee Cardiovascular Medical and Research Center in Tehran, Iran. She completed her subspecialty training in echocardiography and focuses on valvular heart disease, right-sided cardiac pathology, and multimodality cardiovascular imaging. Her clinical and research interests include infective endocarditis, pulmonary valve disorders, and imaging-guided decision-making in complex structural heart conditions. As the lead author of this case report, she oversaw the acquisition and interpretation of imaging data and contributed to the clinical analysis and manuscript preparation.

## Data Availability

The data underlying this article are not publicly available due to patient confidentiality and ethical restrictions. Data may be made available from the corresponding author upon reasonable request.

## References

[1] Habib G, Lancellotti P, Antunes MJ, Bongiorni MG, Casalta JP, Del Zotti F, et al 2015 ESC guidelines for the management of infective endocarditis: the task force for the management of infective endocarditis of the European Society of Cardiology (ESC). endorsed by: European association for cardio-thoracic surgery (EACTS), the European association of nuclear medicine (EANM). Eur Heart J 2015;36:3075–3128. 10.1093/eurheartj/ehv319 26320109

[2] Baddour LM, Wilson WR, Bayer AS, Fowler VG Jr, Tleyjeh IM, Rybak MJ, et al Infective endocarditis in adults: diagnosis, antimicrobial therapy, and management of complications: a scientific statement for healthcare professionals from the American Heart Association. Circulation 2015;132:1435–1486. 10.1161/CIR.0000000000000296 26373316

[3] Iung B, Vahanian A. Epidemiology of valvular heart disease in the adult. Nat Rev Cardiol 2011;8:162–172. 10.1038/nrcardio.2010.202 21263455

[4] Ansari MJ, Rashid OB, Shanbhag V, Ghori UA, Bandyopadhyay D. Isolated native pulmonary valve endocarditis: a case report and review of the current literature. J Cardiothorac Surg 2021;16:218.

[5] Feuchtner GM, Stolzmann P, Dichtl W, Plank F, Muigg K, Friedrich G, et al Multimodality imaging in infective endocarditis: comprehensive evaluation by echocardiography and cardiac computed tomography. Heart 2020;106:1506–1513.

[6] Habets J, Symersky P, de Mol BAJM, de Vries JP, Budde RPJ, Legemate DA, et al Diagnostic evaluation of infective endocarditis: current status and future directions. Int J Cardiovasc Imaging 2019;35:1425–1438.

[7] Gagnier JJ, Kienle G, Altman DG, Moher D, Sox H, Riley D, et al The CARE guidelines: consensus-based clinical case report guideline development. J Clin Epidemiol 2014;67:46–51. 10.1016/j.jclinepi.2013.08.003 24035173

